# Major Artery Occlusion: a Rare Complication of Sickle Cell Disease

**DOI:** 10.4084/MJHID.2010.007

**Published:** 2010-05-04

**Authors:** Adnan Agha, Mohammad Al-Hakami, Ghulam Shabbir

**Affiliations:** Department of Internal Medicine, King Fahad Hospital, Armed Forces Hospital Program Southern Region, Khamis Mushyt, P.O. Box 101, Kingdom of Saudi Arabia.

## Abstract

Sickle cell disease is hereditary hemoglobinopathy which causes haemolytic anemia, vaso-occlusive crisis, ischemic injuries and many other morbidities like cerebral infarction. In this report, we describe a case of a young patient with sickle cell disease presenting with right-sided weakness and slurring of speech with examination confirming right-sided hemiparesis with motor aphasia. On further investigation, she was found to have frontotemporal infarction. On magnetic resonance imaging with angiography, she was found to have absent circulation in left internal carotid artery probably secondary to sickle cell disease. Major vessel occlusion is rare complication of sickle cell disease that one must bear in mind.

## Introduction:

Sickle cell disease is the most common genetic hemoglobinopathy diseases characterized by hemolytic anemia and morbidities like painful vaso-occlusive crisis and ischemic injury. It also predisposes to cerebral infarcts in relatively younger age. Even rarely sickle cell disease may result in vessel occlusion. In this case report we attempt to describe a rare case of major vessel occlusion in a young patient with known sickle disease.

## Case Report:

A 22-year-old Saudi female presented to Emergency Department with a 2-day history of right-sided weakness and slurring of speech which was sudden in onset. Her past medical history was significant for ischemic stroke in the left frontal region 2 years back and for having sickle cell disease with frequent visits to hospital for vaso-occlusive crisis. She was on regular frequent blood transfusions with last transfusion over three months ago. Since her ischemic stroke, the patient had been on aspirin 81 mg daily. She was vaccinated against Streptococcus pneumoniae, Haemophilus influenzae type B, Neisseria meningitidis, with no indications of iron overload, taking hydroxyurea 500 mg daily and was being regularly followed up in our sickle cell clinic. She had no history of smoking or illicit drug use. The patient was married with one kid but she was not taking any oral contraceptives.

Examination revealed right-sided hemiparesis with motor aphasia. Her laboratory data is as shown in the table. CT scan showed frontotemporal ischemic changes suggestive of infarction. Suspicion for cortical venous thrombosis in light of sickle cell disease and predisposing thrombophilic tendency lead to performing an MRI Brain with angiography that revealed near absent circulation in left internal carotid artery.

## Discussion:

Occlusion of a major vessel like carotid artery is a rare complication of sickle cell disease which has been reported before.^1^ Twenty-four percent of SCA patients suffer a stroke by the age of 45 which carries significant morbidity and mortality.^2^ In Saudi sickle cell population the presence of the Mediterranean G6PD mutation (S188F) predisposes more to cerebral stroke.^3^

Sickle cell disease is an inherited disorder of hemoglobin that is among the most common genetic diseases in the world. It is characterized by lifelong hemolytic anemia and many other significant morbidities largely related to painful and debilitating vaso-occlusive phenomenon.^4^ Sickle cell disease (SCD) was first described in 1910, in a dental student who presented with pulmonary symptoms.^5^ Herrick coined the term “sickle-shaped” to describe the peculiar appearance of the red blood cell of this patient. However, given the patient’s symptoms, he was not sure at the time whether the blood condition was a disease sui generis or a manifestation of another disease.^6^ Ingram and colleagues demonstrated shortly thereafter that the mutant sickle hemoglobin (Hb S) differed from normal hemoglobin A by a single amino acid.^7^ This was followed by studies that analyzed the structure and physical properties of Hb S, which formed intracellular polymers upon deoxygenation.^8^ The clinical manifestations of sickle cell anemia (SCA) result primarily from hemolytic anemia and the effects of repeated intravascular sickling, causing vasoocclusion and ischemic injury while lower levels of fetal hemoglobin (Hb F) and higher white blood cell (WBC) counts are associated with an increased incidence of SCA-related events, organ damage, and mortality.^9^

The chronic organ damage in SCA is an insidious process that may affect almost every organ system and can lead to considerable morbidity and mortality at an early age including loss of splenic function,^10^ sickle nephropathy (proteinuria and renal insufficiency),^11^ pulmonary hypertension,^12^ and brain ischemic lesions,^13^ are examples of long-term end-organ damage observed in SCA. The Stroke Prevention Trial in Sickle Cell Anemia (STOP) trial has demonstrated that the risk of stroke due to SCD is 10% per year in children not undergoing transfusion and 1% per year in children undergoing regular blood transfusion.^14^,^15^

## Conclusion:

The idea behind this manuscript is to highlight that major vessel occlusion causing stroke in patients with sickle cell disease is a possibility which should be kept in mind by emergency physicians.

## Figures and Tables

**Figure 1.**
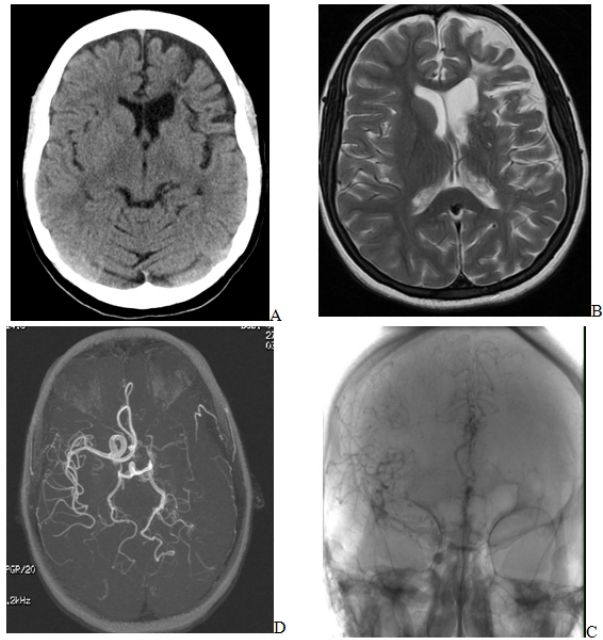
(A to D) with figure 1A showing ischemic changes in left frontoparietal region on CT scan brain plain, with figure 1B showing similar findings on T2 FLAIR MRI brain with enhancement. Figure 1C showing absence of arterial supply in the left MCA territory on MR angiogram while figure 1D showing absence of left internal carotid blood supply due to internal carotid occlusion.

**Table 1:** Laboratory Data of the Patient

Hemoglobin	7.8 gm/dl	Urea	4.2 mmol/L
White blood cells	16.4	Creatinine	67 mmol/L
Platelets	451	Alanine aminotranferase	41 IU/L
Hemoglobin S	76%	Bilirubin	44 mmol/L
